# Enhancing Patient Safety Through Improved Discharge Documentation: A Quality Improvement Audit at Hasahesa Teaching Hospital, Sudan

**DOI:** 10.7759/cureus.96230

**Published:** 2025-11-06

**Authors:** Mustafa Mohamed, Abdelrahman Elfatih Elsheikh Abdelrahim, Elaf Abdelrahman, Lamees Ahmed, Maisoon Musa, Abd elazim Mohamed, Somia Elhag, Sara Elhadi, Nassma Ibrahim, Rawan Alfaqir, Randa Ahmed, Ola Himmed, Thekra Mohamed, Ahmed Saeed, Abdalrahman Saeed, Zeinab Osman, Mohamed Alsayed, Maram Babiker, Douaa Ibrahim, Eithar Babike, Amro Osama Fathi Mohammed

**Affiliations:** 1 General Surgery, Prince Othman Digna Teaching Hospital, Port Sudan, SDN; 2 Medicine, Almanagil Teaching Hospital, Almanagil, SDN; 3 Internal Medicine, Al Neelain University, Khartoum, SDN; 4 General Practice, Al Neelain University, Khartoum, SDN; 5 Emergency, Hasahesa Teaching Hospital, Al-Hasaheisa, SDN; 6 General Medicine, Al Neelain University, Khartoum, SDN; 7 Medical Education, Al Neelain University, Khartoum, SDN; 8 Dentistry, Hasahesa Teaching Hospital, Al-Hasaheisa, SDN; 9 General Surgery, Al Neelain University, Khartoum, SDN; 10 Emergency Medicine, Hasahesa Teaching Hospital, Al-Hasaheisa, SDN

**Keywords:** clinical audit, continuity of care, discharge documentation, patient safety, quality improvement

## Abstract

Background: Effective discharge documentation is essential for ensuring continuity of care and patient safety. In many resource-limited hospitals, discharge summaries are often incomplete, leading to communication gaps, preventable errors, and avoidable readmissions. This audit evaluated the impact of introducing a standardized discharge card on the quality of discharge documentation at Hasahesa Teaching Hospital, Sudan.

Methods: A two-cycle clinical audit was conducted in the Internal Medicine Department. The baseline review (Cycle 1) assessed 50 discharge cards retrospectively over a two-week period (20 March to 2 April 2025) using a structured checklist adapted from international standards. Following this, a one-month intervention was implemented, introducing a newly designed discharge card supported by staff training and visual aids. The re-audit (Cycle 2) reviewed 50 discharge cards over three months (3 May-2 August 2025). Compliance across multiple documentation domains was compared using the chi-square test.

Results: Documentation improved significantly across all domains after the intervention. Recording of demographic details, clinical information, treatments, discharge diagnosis, and follow-up instructions rose from baseline levels of 22-56% to post-intervention levels of 94-100%, representing absolute improvements of 38-72 percentage points (95% CIs provided in the full text). All improvements were statistically significant (p < 0.001). Staff feedback indicated that the standardized card was clear, practical, and enhanced workflow.

Conclusion: The standardized discharge card markedly improved the quality and completeness of discharge documentation, providing a simple, low-cost, and effective intervention to strengthen patient safety and continuity of care in a resource-limited setting. Wider implementation and longer-term evaluation are recommended to ensure sustainability and scalability.

## Introduction

At the time of hospital discharge, a patient's inpatient treatment comes to an end, and their continued medical management is handed off to primary care, community agencies, or even their own home. To guarantee continuity of care and patient safety during this transition, precise, up-to-date, and thorough documentation is essential. Multiple departments must work together to ensure a smooth discharge because of the inherent complexity of the procedure [1]. During this time, patients are at a heightened risk of problems related to healthcare quality and safety caused by incomplete or incorrect information, which can result in treatment delays or omissions, unnecessary effort, or even adverse events. Among the 19% of patients who encountered adverse effects after discharge, 31% were deemed avoidable, according to one study [2,3].

Research by Hoyer et al. [4] demonstrated that delayed completion of discharge reports was associated with higher patient readmission rates. This underscores the importance of discharge documentation that is patient-centered, educational, and effective, as it may be sent directly to primary care providers or carried by patients themselves. The discharge card serves as a critical communication tool between healthcare providers across different facilities.

To ensure safe transitions of care, several strategies have been recommended, including staff education and training, medication reconciliation, standardized discharge protocols, and active involvement of patients and caregivers [5-7]. Among these interventions, structured discharge tools and staff education have shown the strongest evidence for improving patient safety and reducing preventable readmissions, as they directly address communication gaps and ensure protocol adherence [5-10]. Although the adoption of electronic discharge summaries with automated alerts has enhanced workflow in some hospitals, cost and usability challenges limit their widespread use in low-resource environments. In this context, a paper-based discharge card was adopted due to the lack of electronic systems and financial constraints, ensuring feasibility, sustainability, and immediate applicability within the local setting.

The audit team at Soba Center for Audit and Research (SCAR) previously evaluated the quality and content of discharge cards [11]. Their findings indicated that a significant proportion of discharge summaries were not written using the structured pre-designed format, limiting the delivery of valid, relevant, and sufficient health information. The current structured format was not sufficient to improve summary quality [11].

Building on these insights and guidance from the World Health Organization [12], we implemented a newly designed discharge card at Hasahesa Teaching Hospital to enhance the completeness and accuracy of discharge documentation. This study aimed to evaluate the impact of these interventions by comparing the quality of the new discharge cards to the previous format.

## Materials and methods

Study design and setting

This quality improvement project was conducted in two audit cycles: a retrospective baseline review (Cycle 1) and a prospective re-audit (Cycle 2). The study took place in the Internal Medicine Department of Hasahesa Teaching Hospital, a 150-bed tertiary referral center in Sudan that serves both urban and rural populations. The department was selected due to its high discharge volume and previously recognized deficiencies in discharge documentation.

Problem identification and rationale

**Figure 1:**
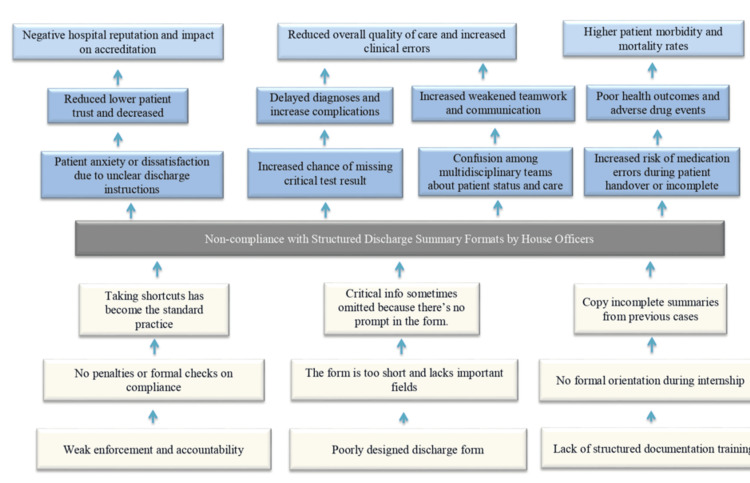
Problem Tree Analysis of Inadequate Discharge Documentation Problem tree analysis illustrating the root causes, underlying systemic weaknesses, and consequences of poor discharge documentation at Hashesa Teaching Hospital. Key issues included poorly designed forms, lack of structured training, weak enforcement, and a culture of shortcuts, leading to communication breakdowns, increased clinical errors, and adverse patient outcomes.

Prior to the audit, discharge summaries were often incomplete or inconsistently documented, which compromised communication between hospital teams, primary care physicians, and patients. Informal feedback from clinicians revealed several challenges, including the absence of a standardized discharge template, variability in individual recording practices, and limited awareness of the clinical and medico-legal importance of comprehensive discharge documentation. To systematically explore these factors, a problem-tree analysis was conducted during a structured one-hour session involving three physicians and two nurses from the Internal Medicine Department. Root causes were identified based on Cycle 1 audit findings and informal staff interviews. This process enabled categorization of contributing factors into system-level weaknesses (e.g., lack of policy and template), staff-level issues (e.g., awareness, training, and workload), and process-level inefficiencies (e.g., lack of accountability for medication reconciliation). A visual representation of the analysis and further detail is provided in Appendix 1 (Figure 1).

Team and stakeholder engagement

The QI team included three internal medicine physicians (one registrar and two medical officers) as audit leads, a senior charge nurse, and the ward clerk supervisor. Departmental leadership formally endorsed the project and approved the discharge card as the standard form for routine use beyond the study period. Stakeholder engagement was prioritized to enhance sustainability: junior doctors and nursing staff collaborated in co-designing the card to ensure clinical usability, while clerical staff ensured compatibility with existing documentation workflows. A written policy update was issued to mandate the continued use of the finalized discharge card.

Baseline measurement (Cycle 1)

**Figure 2:**
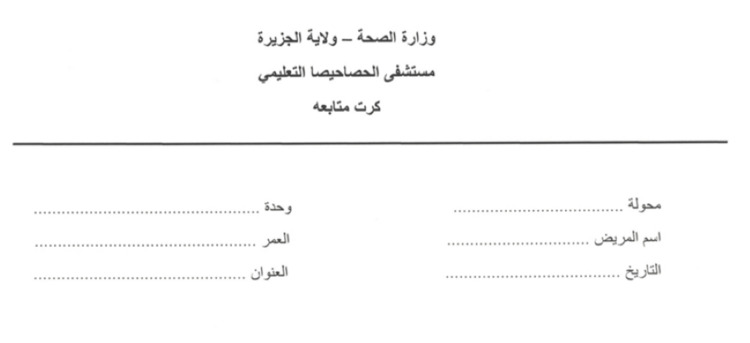
Previous Discharge Card Used in the Internal Medicine Department The older discharge card used prior to the intervention. It contained only limited fields for patient demographics and referral information, lacking structured sections for clinical details, treatment records, discharge instructions, and follow-up, contributing to incomplete documentation and communication gaps. Follow-up Card (original form in Arabic) English translation: Ministry of Health – Gezira State Al-Hasahesa Teaching Hospital Follow-up Card Referral: ………………… Patient name: ………………… Date: ………………… Unit: ………………… Age: ………………… Address: …………………

The baseline assessment (Cycle 1) was carried out between 20 March and 2 April 2025, covering a consecutive two-week period. Fifty discharge cards were selected via simple random sampling from the department’s discharge list of all eligible patients during this timeframe. A structured audit checklist, adapted from international recommendations on safe hospital discharge and tailored to the local context, was used for evaluation (Appendix 2, Figure 2). Each parameter was scored as present or absent. Three physicians independently reviewed the cards, demonstrating substantial inter-rater reliability (Fleiss’ κ = 0.82). Discrepancies were resolved by consensus. The baseline results confirmed widespread deficiencies, with particularly low completion rates in medication reconciliation and follow-up instructions.

Intervention

**Figure 3:**
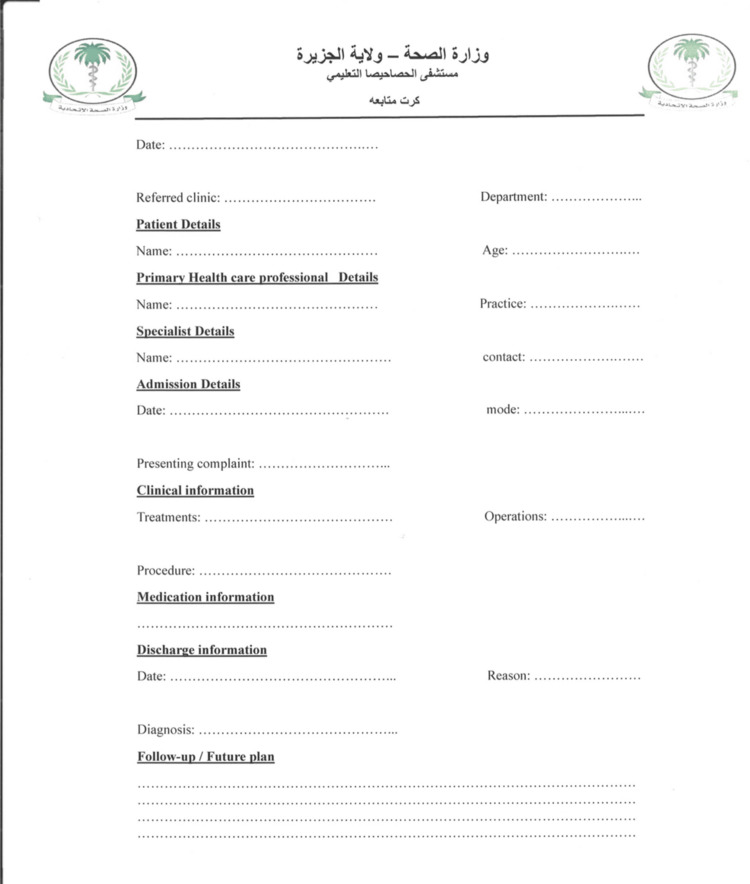
Newly Designed Standardized Discharge Card The standardized discharge card introduced at Hashesa Teaching Hospital as part of the intervention phase. The card includes clearly defined sections for patient identifiers, clinical details, treatment summaries, medication information, discharge details, and follow-up plans, aiming to improve completeness and accuracy of documentation [10].

The intervention phase took place between 3 April and 2 May 2025. During this period, a standardized discharge card was designed and implemented. The card contained structured sections for essential documentation elements, including patient identifiers, clinical summary, diagnosis, treatment, medication reconciliation, and follow-up arrangements (Appendix 3, Figure 3). Staff were introduced to the new card through formal training sessions that combined short lectures, demonstrations, and case-based exercises, with emphasis on accuracy and medication safety. Visual job aids were placed in clinical areas, and biweekly feedback huddles were organized to troubleshoot early issues. Key refinements during iterative testing included (1) adding explicit prompts for medication reconciliation, (2) reordering sections to improve workflow, (3) enlarging the free-text space for clinical summaries, and (4) bold labeling of follow-up instructions to improve visibility. The finalized discharge card (version 1.0), which was used in the re-audit, has been added as a high-quality figure to facilitate adoption by other institutions.

Re-measurement (Cycle 2)

The re-audit (Cycle 2) was conducted between 3 May and 2 August 2025, covering a three-month period immediately after the intervention. All eligible discharge cards during this period were included (total coverage, n = 50), which also helped minimize a Hawthorne effect by extending data collection beyond the initial post-training weeks. The same checklist and scoring criteria were used to ensure comparability. To limit assessment bias, reviewers were blinded to whether cards belonged to Cycle 1 or Cycle 2 during evaluation and scored them independently before consensus review. In addition to quantitative measurements, informal staff feedback was collected to explore perceptions of the discharge card’s usability, workflow integration, and communication impact.

Outcome measures

The primary outcome was the proportion of discharge summaries that were complete across each predefined documentation domain. A domain was considered complete only when all required fields within that domain were documented. In addition, a composite measure of “fully complete discharge card” was calculated, defined as documentation of all domains without any missing items. Secondary measures included qualitative staff feedback on usability, workflow efficiency, and communication during discharge.

Data analysis

Data from both cycles were entered into a structured database and analyzed using Statistical Product and Service Solutions (SPSS, version 29; IBM SPSS Statistics for Windows, Armonk, NY). Frequencies and percentages were calculated for each documentation parameter. Comparisons between baseline and re-audit results were conducted using the chi-square test or Fisher’s exact test where expected counts were small. Absolute risk differences with corresponding 95% confidence intervals were additionally computed to quantify the magnitude of improvement for each domain. Given the multiple endpoints evaluated, interpretation of statistical significance considered the potential for type I error inflation, and results are presented with this context. A p-value of less than 0.05 was considered statistically significant.

Ethical considerations

Ethical approval for the project was obtained from the Institutional Review Board of Hasahesa Teaching Hospital (HTH/IRB/2025/047). All patient identifiers were removed before data entry to ensure confidentiality. Given that the project was a quality improvement initiative with no direct patient interventions, the requirement for individual informed consent was waived.

## Results

The audit demonstrated a substantial improvement in documentation practices across all assessed parameters following the intervention (Table 1). In the first cycle, compliance with recording essential patient details such as the full name (13, 26%) was markedly low, but this increased to 47 (94%) in the second cycle (χ² = 48.17; p < 0.001). Similarly, documentation of age improved from 28 (56%) in the first cycle to 50 (100%) in the second (χ² = 28.21; p < 0.001).

**Table 1:** Improvements in Documentation Compliance Between the First and Second Audit Cycles This table presents the comparison of documentation practices across different domains during the first audit cycle (n=50) and second audit cycle (n=50). Compliance is reported as frequency (percentage). The chi-square test was used to compare differences between the two cycles, and p-values <0.05 were considered statistically significant.

Parameter Category	Parameter	First Cycle (n=50)	Second Cycle (n=50)	Chi-square	P-value
Patient Details	Full Name	13 (26%)	47 (94%)	48.17	<0.001
	Age	28 (56%)	50 (100%)	28.21	<0.001
Hospital Details	Hospital Name	28 (56%)	50 (100%)	28.21	<0.001
	Department	25 (50%)	50 (100%)	33.33	<0.001
Specialist Details	Specialist Name	26 (52%)	50 (100%)	31.58	<0.001
Primary Healthcare Provider	Doctor Name	16 (32%)	50 (100%)	51.52	<0.001
	Practice / Clinic	14 (28%)	50 (100%)	56.25	<0.001
Admission Details	Admission Date	25 (50%)	50 (100%)	33.33	<0.001
	Admission Mode (Emergency / Referral / Elective)	11 (22%)	48 (96%)	56.59	<0.001
	Presenting Complaint	29 (58%)	50 (100%)	26.58	<0.001
Clinical Information	Clinical Summary	23 (46%)	50 (100%)	36.99	<0.001
	Diagnoses	27 (54%)	50 (100%)	29.87	<0.001
Treatments / Procedures	Details of Procedures	18 (36%)	48 (96%)	40.11	<0.001
	Treatments Administered	23 (46%)	49 (98%)	33.53	<0.001
	Medication Information	24 (48%)	50 (100%)	35.14	<0.001
Discharge Information	Discharge Date	23 (46%)	49 (98%)	33.53	<0.001
	Reason for Discharge (Recovery / Transfer)	17 (34%)	50 (100%)	49.25	<0.001
	Discharge Diagnosis	13 (26%)	50 (100%)	58.73	<0.001
	Signature	12 (24%)	47 (94%)	50.64	<0.001
Follow-Up / Future Management	Follow-Up Plan / Recommendations	20 (40%)	50 (100%)	42.86	<0.001
	Next Appointment Date / Recommended Review	25 (50%)	50 (100%)	33.33	<0.001

Hospital details also showed significant improvement. Recording of hospital name rose from 28 (56%) to 50 (100%) (χ² = 28.21; p < 0.001), and department documentation improved from 25 (50%) to 50 (100%) (χ² = 33.33; p < 0.001).

Specialist details demonstrated a similar trend, with specialist name documentation improving from 26 (52%) to 50 (100%) (χ² = 31.58; p < 0.001). Information on the primary healthcare provider, particularly the doctor’s name, increased from 16 (32%) to 50 (100%) (χ² = 51.52; p < 0.001), while practice or clinic details improved from 14 (28%) to 50 (100%) (χ² = 56.25; p < 0.001).

Admission-related data also improved considerably. Admission date documentation increased from 25 (50%) to 50 (100%) (χ² = 33.33; p < 0.001). The mode of admission, which was poorly documented at baseline 11 (22%), rose to 48 (96%) (χ² = 56.59; p < 0.001).

Clinical information showed substantial gains. Recording of presenting complaints increased from 29 (58%) to 50 (100%) (χ² = 26.58; p < 0.001), clinical summaries from 23 (46%) to 50 (100%) (χ² = 36.99; p < 0.001), and diagnoses from 27 (54%) to 50 (100%) (χ² = 29.87; p < 0.001).

Documentation of treatments and procedures improved significantly. Recording of procedure details rose from 18 (36%) to 48 (96%) (χ² = 40.11; p < 0.001). Treatments administered increased from 23 (46%) to 49 (98%) (χ² = 33.53; p < 0.001), and medication information from 24 (48%) to 50 (100%) (χ² = 35.14; p < 0.001).

Discharge information also showed notable progress. Discharge date documentation increased from 23 (46%) to 49 (98%) (χ² = 33.53; p < 0.001), reason for discharge from 17 (34%) to 50 (100%) (χ² = 49.25; p < 0.001), discharge diagnosis from 13 (26%) to 50 (100%) (χ² = 58.73; p < 0.001), and signature from 12 (24%) to 47 (94%) (χ² = 50.64; p < 0.001).

Finally, follow-up and future management details improved to complete compliance. The follow-up plan or recommendations rose from 20 (40%) to 50 (100%) (χ² = 42.86; p < 0.001), and the next appointment date from 25 (50%) to 50 (100%) (χ² = 33.33; p < 0.001). Overall completeness of discharge documentation improved markedly, with the proportion of fully complete discharge cards increasing from 2 (4%) in Cycle 1 to 41 (82%) in Cycle 2 (χ² = 54.17; p < 0.001).

## Discussion

This audit demonstrated that the implementation of a standardized discharge card, supported by structured staff training, substantially improved the completeness and accuracy of discharge documentation at Hasahesa Teaching Hospital. Prior to the intervention, essential information such as demographic details, clinical summaries, treatment records, and follow-up plans was inconsistently documented, exposing patients to potential gaps in care during transition. Following the introduction of the new card, compliance rose markedly across all domains, with several parameters achieving 100% documentation. These improvements likely contribute to safer transitions of care, though downstream clinical outcomes were not directly measured. Future studies could explore the relationship between improved discharge documentation and outcomes such as readmissions, medication discrepancies, or adverse events to further evaluate clinical impact.

The findings resonate with earlier studies that have highlighted the dangers associated with poor discharge communication. Forster et al. showed that nearly one in five patients experienced an adverse event following discharge, with one-third considered preventable through better documentation and communication [2]. Similarly, Kripalani et al. emphasized that deficits in information transfer between hospital and primary care physicians were a major threat to patient safety [3]. The improvements seen in this audit suggest that targeted interventions, even in resource-limited settings, can bridge these critical gaps.

The timeliness of discharge summaries is equally important. Research by Hoyer et al. found that delays in completing discharge reports were directly associated with increased readmission rates, highlighting that effective communication is not only about completeness but also about prompt delivery [4]. More recently, Shpigel et al. demonstrated that a structured quality improvement initiative improved the rate of discharge summary completion within 48 hours from 48% to 87.1% [13]. These findings reinforce that setting clear timeliness goals and applying structured interventions can yield significant gains in discharge practice.

The results also align with guidance from professional organizations. The Royal College of Nursing stresses the importance of structured discharge planning to ensure smooth transitions and avoid fragmented care [5]. Similarly, the Royal College of Physicians advocates for standardized medical record-keeping formats, which have been shown to improve physician performance and patient outcomes [10]. The remarkable improvements in compliance observed in this audit provide local evidence that such standards are both achievable and effective when adapted to the realities of low-resource hospitals. Initial implementation challenges - such as periodic staff turnover, limited paper supply, and variable adherence early on - were mitigated through ongoing induction training for new staff, centralized printing and distribution of the discharge card, and routine monitoring with feedback to reinforce use.

Electronic discharge systems have demonstrated further gains in efficiency and safety in high-income countries. Dalal et al., for example, reported that computerized applications for pending tests at discharge improved workflow and reduced errors [8]. However, challenges with cost, infrastructure, and usability limit widespread adoption in many low- and middle-income settings. In this context, the paper-based discharge card implemented in Hashesa provides a feasible, low-cost alternative that achieves significant gains without requiring extensive technological investment. Mathiesen et al. also showed that integrated medicines management improves the quality of medication-related content in discharge summaries [14], which may be an area for future enhancement in our system.

The present findings build on local evidence from Sudan, where a previous evaluation at Soba University Hospital found that discharge summaries were often incomplete and failed to follow structured formats [11]. By demonstrating that a redesigned, context-specific card combined with staff engagement can produce rapid improvements, this audit contributes valuable evidence on how to strengthen transitional care in resource-constrained environments. Furthermore, strategies that simplify discharge instructions, as reported by DeSai et al. [15], could further improve patient comprehension and adherence when combined with structured templates.

Communication interventions at discharge have broader impacts beyond documentation alone. Becker et al. showed that such interventions are significantly associated with fewer readmissions, higher adherence, and greater patient satisfaction across many settings [16]. This suggests that improving discharge communication is not just a matter of administrative completeness but has real health outcomes. Our audit’s improvements in documentation may thus translate into better patient safety, treatment continuity, and possibly lower readmission rates, if sustained and coupled with measures to improve patient understanding.

Implications for practice

The improvements observed in this audit highlight the practical value of structured discharge tools in resource-constrained hospitals. By standardizing documentation, the intervention reduced variability in practice and ensured that essential patient information was consistently recorded. This approach directly supports safer care transitions, minimizing the risk of treatment delays, omissions, or avoidable readmissions. Evidence from the Royal College of Nursing emphasizes that structured discharge planning should be a routine part of clinical practice to maintain continuity of care across services [5]. In low-resource environments where electronic medical records are not yet feasible, a paper-based discharge card provides an accessible and sustainable solution. Scaling this model to other departments or hospitals could strengthen overall patient safety systems in Sudan and similar contexts. A structured expansion roadmap is proposed: (1) development of a reproducible training package for new staff, (2) designation of a responsible clinical lead and ward clerk to oversee ongoing card utilization, and (3) quarterly audit cycles for continuous performance monitoring. Key performance indicators (KPIs) may include sustaining ≥95% completeness across all core documentation domains for at least six months, staff satisfaction with usability, and timely dissemination of discharge summaries to receiving providers. Simplification of instructions for patients and better integration with medicines management may further enhance the utility of discharge summaries, as recent evidence demonstrates [13-15].

Strengths and limitations

A key strength of this project lies in its pragmatic design, which combined a locally tailored intervention with active staff involvement. The use of a closed-loop audit structure within a single Plan-Do-Study-Act (PDSA) cycle allowed iterative refinement and evaluation of the intervention. The statistically significant gains across nearly all documentation domains provide objective evidence of effectiveness. Moreover, the intervention was low-cost, aligned with available resources, and therefore feasible for adoption in similar low-resource hospitals. The PDSA structure followed four phases: baseline gap identification and card development (Plan), staff training and implementation (Do), performance evaluation through re-audit (Study), and hospital-wide adoption supported by a policy update (Act). A summary of this process is presented in Appendix 4 (Table 2).

Nevertheless, certain limitations must be acknowledged. The audit was confined to a single department, which may limit generalizability to other specialties or institutions. The relatively small sample size (50 discharge cards per cycle) restricts the breadth of conclusions, though the improvements were large and consistent. The short follow-up period did not allow assessment of long-term sustainability, and documentation improvements may diminish without continued reinforcement. A third audit cycle is scheduled approximately 12 months post-intervention to assess whether improvements are sustained over time and to determine if further booster training or refinements are required. Additionally, the project assessed documentation completeness as a process measure but did not evaluate patient comprehension of discharge instructions. Future cycles could incorporate patient-centered measures such as comprehension checks (e.g., teach-back technique) or readability assessment of written instructions to better link documentation quality with patient outcomes. Initial implementation challenges - including periodic staff turnover and maintenance of adequate paper supply - were mitigated through induction training for new staff, centralized printing, and routine feedback mechanisms to reinforce adherence.

## Conclusions

This audit demonstrated that introducing a standardized discharge card with staff training significantly improved the completeness and accuracy of discharge documentation at Hasahesa Teaching Hospital. The intervention addressed deficiencies in demographic, clinical, treatment, and follow-up information, ensuring safer patient transitions. As a low-cost and feasible strategy, it highlights the value of structured tools in resource-limited settings. Broader implementation across other departments and hospitals could amplify its impact. Sustained monitoring, patient comprehension, and integration with digital solutions remain important for long-term success, with quarterly re-audits, refresher training for staff during onboarding, and oversight by the ward clerk supervisor to maintain adherence and performance. This project demonstrates that, with a systematic, low-cost approach, resource-limited hospitals can achieve excellence in discharge documentation, establishing a strong foundation for safer care transitions and future integration with digital health systems.

## Appendices

**Table 2:** Summary of the Plan–Do–Study–Act (PDSA) Cycle Used to Improve Discharge Documentation at Hashesa Teaching Hospital This table outlines the single PDSA cycle applied in this quality improvement project. The “Plan” phase included baseline gap identification and development of the improved discharge card. The “Do” phase involved staff training and pilot implementation. The “Study” phase assessed documentation completeness through the re-audit and gathered staff feedback. The “Act” phase consisted of refining and institutionalizing the intervention via departmental policy approval to ensure sustainability.

PDSA Cycle	Aim	Key Activities	Result
Cycle 1	Identify baseline gaps & provide training	Audit Cycle 1, staff teaching sessions	Awareness increased
Cycle 2	Improve card design & reinforce use	Card redesign, feedback huddles	Compliance improved

## References

[1] (2025). Social care online. London Station Off.

[2] Forster AJ, Murff HJ, Peterson JF, Gandhi TK, Bates DW (2003). The incidence and severity of adverse events affecting patients after discharge from the hospital. Ann Intern Med.

[3] Kripalani S, LeFevre F, Phillips CO, Williams MV, Basaviah P, Baker DW (2007). Deficits in communication and information transfer between hospital-based and primary care physicians: implications for patient safety and continuity of care. JAMA.

[4] Hoyer EH, Odonkor CA, Bhatia SN, Leung C, Deutschendorf A, Brotman DJ (2016). Association between days to complete inpatient discharge summaries with all-payer hospital readmissions in Maryland. J Hosp Med.

[5] (2025). Ready to go? Planning the discharge and the transfer of patients from hospital and intermediate care. Dep Heal.

[6] Waring J, Marshall F, Bishop S (2014). Knowledge sharing across the boundaries between care processes, services and organisations: the contributions to 'safe' hospital discharge. Heal Serv Deliv Res.

[7] Laugaland K, Aase K, Barach P (2012). Interventions to improve patient safety in transitional care -- a review of the evidence. Work.

[8] Dalal AK, Poon EG, Karson AS, Gandhi TK, Roy CL (2011). Lessons learned from implementation of a computerized application for pending tests at hospital discharge. J Hosp Med.

[9] (2025). Generic medical record keeping standards. Clin Med (Northfield. Il). M.

[10] Carpenter I, Ram MB, Croft GP, Williams JG (2007). Medical records and record-keeping standards. Clin Med (Lond).

[11] Dafaalla MD, Abdalrahman I, Kheir A (2014). Adequacy of the discharge summary at Soba university hospital: where are we now?. Online J Clin Audit.

[12] Knai C, Brusamento S, Legido-Quigley H (2013). The role of discharge summaries in improving continuity of care across borders. Eurohealth.

[13] Shpigel E, Shpigel N, Ndoja S (2025). Improving timeliness of patient discharge summaries within orthopaedic surgery: a quality improvement initiative. Open Orthopaedics Journal.

[14] Mathiesen L, Nguyen TB, Dæhlen I, Mowé M, Lea M (2024). Effect of integrated medicines management on quality of discharge medication information - a secondary endpoint in a randomized controlled trial. Int J Qual Health Care.

[15] DeSai C, Janowiak K, Secheli B, Phelps E, McDonald S, Reed G, Blomkalns A (2021). Empowering patients: simplifying discharge instructions. BMJ Open Qual.

[16] Becker C, Zumbrunn S, Beck K (2021). Interventions to improve communication at hospital discharge and rates of readmission: a systematic review and meta-analysis. JAMA Netw Open.

